# Consultation, Consent, and the Silencing of Indigenous Communities

**DOI:** 10.1111/japp.12438

**Published:** 2020-05-25

**Authors:** Leo Townsend, Dina Lupin Townsend

**Affiliations:** ^1^ Department of Philosophy University of Vienna Vienna Austria; ^2^ School of Law University of the Witwatersrand Johannesburg South Africa

## Abstract

Over the past few decades, Indigenous communities have successfully campaigned for greater inclusion in decision‐making processes that directly affect their lands and livelihoods. As a result, two important participatory rights for Indigenous peoples have now been widely recognized: the right to consultation and the right to free, prior and informed consent (FPIC). Although these participatory rights are meant to empower the speech of these communities—to give them a proper say in the decisions that most affect them—we argue that the way these rights have been implemented and interpreted sometimes has the opposite effect, of denying them a say or ‘silencing’ them. In support of this conclusion we draw on feminist speech act theory to identify practices of locutionary, illocutionary, and perlocutionary group silencing that arise in the context of consultation with Indigenous communities.

## Introduction

Since the beginning of colonial times, Indigenous peoples have been given little say in decision‐making processes that directly affect their land and livelihoods, including decisions about natural resource extraction or industrial development on their traditional territory. Historically, this silencing of Indigenous voices and viewpoints has taken the form of *exclusion*. Indigenous communities were never given a seat at the table, never asked to contribute to the relevant discussions—they were *given no say* in the sense that their input was never sought and never permitted.^1^

In the past few decades, however, Indigenous communities, activists, lawyers, scholars and organisations have successfully campaigned for the greater inclusion of Indigenous peoples in these decision‐making processes. As a result, two important participatory rights for Indigenous peoples have now been widely recognized, in both international law^2^ and in the domestic laws of various countries.^3^ These are the *right to consultation*, which entitles affected communities to enter into dialogue with states or companies about proposed developments, and the *right to free, prior, and informed consent* (FPIC), which entitles Indigenous communities to give or withhold their consent to proposed developments on their land.

Although these participatory rights ensure that Indigenous peoples are not silenced in the sense of being *excluded* from decision‐making, we argue that they nonetheless leave Indigenous peoples vulnerable to other, more subtle forms of silencing. To show this, we examine the implementation and interpretation of Indigenous participatory rights through the lens of feminist speech act theory, in which various practices of disempowering speech or ‘silencing’ have been extensively discussed. From this perspective, both the right to consultation and the right to consent can be understood as efforts to empower the speech of Indigenous communities—to *give them a proper say* in developments that will affect them. But the way these rights have been implemented and interpreted, we suggest, often means that these communities are *denied* their say, and are subjected to practices of group silencing.

The article proceeds as follows. In the first part we lay out our theoretical framework. We first explain the basic Austinian distinction between locutionary, illocutionary, and perlocutionary speech acts, then describe how this distinction has been used to illuminate three forms of silencing—locutionary silencing, illocutionary silencing and perlocutionary silencing—to which group speakers, like individual speakers, are susceptible. We then move on to examine three case studies involving Indigenous communities, each of which we think exemplifies a distinctive practice of group silencing: the first a case of locutionary group silencing, the second a case of illocutionary group silencing, and the third a case of perlocutionary group silencing. The article concludes by considering the broader significance—both philosophical and legal—of identifying and characterizing such practices within the framework of silencing.

## Speech Acts and Silencing

### Austin on Locutionary, Illocutionary, and Perlocutionary Acts

In *How to Do Things With Words,* JL Austin tried to draw attention to the fact that speech is action—that to say things is to do things, and there are many kinds of things that can be done with words.^4^ Indeed, Austin thought that on any given speech occasion the speaker typically performs at least three acts with her words: a locutionary act, an illocutionary act, and a perlocutionary act. These three kinds of act correspond to three different senses in which a speaker can be said to ‘have a say’.

One basic sense in which a speaker might be said to ‘have a say’ is just that she is able to produce meaningful utterances: she is able to come out with strings of words that have content. This very minimal sense of speaking is what Austin called the *locutionary act*. For example a speaker might utter ‘There’s a wolf’, where ‘there’ refers to some proximal location and ‘wolf’ to a wolf. The utterance thus has meaningful content, and different locutionary acts can be distinguished on the basis of their having different contents. So saying ‘There’s a wolf’ is a different locutionary act from saying ‘There’s a frog’ since there is a difference in meaningful content between what is said in each.

A rather different sense in which a speaker can ‘have a say’ refers not simply to meaning but to the *communicative* dimension of speech. In this sense, having a say means being able to perform certain distinctively linguistic acts like promising, telling, warning, betting, and so on. These are all examples of what Austin called *illocutionary acts*. According to Austin’s rough characterisation, these are the acts a speaker performs *in* speaking, through the *use* of locutions. For example, someone who utters ‘There’s a wolf’ may therein be *warning* her friend of the nearby wolf, or she may be *telling* her of it, where warning and telling are illocutionary acts. As this example indicates, illocutionary acts cannot be distinguished by their contents alone, as locutionary acts can. Instead, different illocutionary acts are said to have different *forces*: the force of telling, or warning, or promising, etc.

Finally, there is a third sense of ‘having a say’ which goes beyond the communicative dimension to the *consequential* dimension of speech, the sense in which a speaker’s speech accomplishes certain further effects. In this sense to ‘have a say’ is to have one’s speech make some kind of difference, typically on the audience. To have a say in this sense is to perform what Austin called *perlocutionary acts*. For example, when the speaker warns her friend of the wolf she might persuade her friend to flee; or if she is simply telling her friend of the wolf, then she might convince her friend there’s a wolf, where these things—persuading or convincing—are perlocutionary acts. In contrast to the (illocutionary) things that are done *in* speaking, Austin roughly characterised perlocutionary acts as things done *by* speaking.

This distinction between these three kinds of speech acts—the locutionary, illocutionary, and perlocutionary—does not tell us all that much about the agents who perform those acts, nor does it detail the conditions that need to obtain in order for those agents to successfully ‘have their say’ in these three ways. Given that we shall be focusing on collective speakers and the obstacles they face in successfully having their say, these are important questions for the purposes of this article. In particular, we need to ask: what demands does the successful performance of locutionary, illocutionary and perlocutionary acts place on speakers and their audiences?

Let’s start with locutionary acts. Given that a locutionary act is simply the performance of a meaningful utterance, it would seem that a speaker needs to marshal her linguistic know‐how, and make the right noises or the right signs that will count, according to the conventions in the relevant linguistic community, as performing such an utterance. She needs to be able to come out with words that are hers, and that mean something. That is what it takes on the speaker’s side. As for the audience, there is no requirement for audiences to attend to the speaker’s speech, in order for locutionary acts to be performed. Instead, the requirements on the audience are strictly negative: put simply, the audience should not interfere with the speaker—he should allow her to have her say. This means, inter alia, not jeopardising her opportunity to speak, not speaking over her, not interrupting, and so on. Beyond this, there is no essential role for audiences to play in the successful performance of locutionary acts; indeed, locutionary acts can be performed without so much as being addressed to an audience, let alone heard and understood by one.

Things are very different when it comes to illocutionary acts, at least according to one influential way of developing Austin’s basic framework, which emphasizes the communicative character of illocutionary acts like warning, telling, promising and so on.^5^ According to this approach, one of the key things that is required on the speaker’s side for the successful performance of an illocutionary act, is a certain kind of *communicative intention* in speaking. So one makes an utterance, or locutionary act, with the intention to therein do some illocutionary thing like telling or warning or promising. But uttering a suitable locution (‘There’s a wolf’) with a suitable intention (the intention to warn) is still not enough for the successful performance of illocutionary acts. Because these acts are essentially communicative, their success depends not only on the speaker but also on the audience’s reception of the speaker’s utterances. More specifically, for these acts to be successful, the audience must recognise the communicative intentions of the speaker—recognize which illocutionary thing she means to do with her words. Austin calls this recognition ‘uptake’:

I cannot be said to have warned an audience unless it hears what I say and takes what I say in a certain sense […]. So the performance of an illocutionary act involves the securing of uptake.^6^

Finally, when it comes to perlocutionary acts, the requirements on the speaker and the audience are akin to the requirements in place when an agent performs a physical action. That is, in order to successfully do some perlocutionary thing like persuade, amuse, or convince, a speaker must intend to bring about some effect on the audience, and the audience, if the act is to be successful, must comply. So here too there is a role for both speaker and audience in the successful performance of a perlocutionary act, although the requirements on the audience are rather different from the illocutionary case, since the audience need not recognise what the speaker is intending. I can, for instance, successfully amuse you without your realizing that amusing you is what I intended to do (likewise for other perlocutionary acts like seducing and persuading). So the role of the audience is not to give uptake but to actually be affected in the way the speaker intends. My attempt to persuade you succeeds only if it gets you to change your mind; my attempt to amuse you succeeds only if you find what I say funny (or otherwise amusing), and so on.

### Silencing in an Austinian Framework

This distinction between three ways of ‘having a say’—the locutionary, illocutionary and perlocutionary—has been used to highlight three different ways in which speech can be impeded.^7^ The basic idea is that just as competent speakers are usually in a position to do locutionary, illocutionary and perlocutionary things with words, so too can they be unjustly prevented from doing these things—they can be *denied their say* or ‘silenced’.^8^

The first kind of silencing is locutionary in character. We saw that to perform a locutionary act was to ‘have one’s say’ in the most minimal sense of coming out with meaningful words. But although this is often an easy feat for competent speakers, it can in certain circumstances be made difficult, even impossible. Perfectly competent speakers can be prevented from performing meaningful utterances in all manner of ways, including being physically smothered, being intimidated, being interrupted, being denied the opportunity to speak in one’s own language, or by being usurped—by having one’s opportunity for speech taken up by someone else. When any of these things happen, the speaker’s normal ability to perform meaningful utterances is unjustly impeded. She does not get to have her say in even the most basic, locutionary sense.^9^

The second sort of silencing is ‘illocutionary disablement’.^10^ This is the kind that has received by far the most attention in the literature, notably from Jennifer Hornsby and Rae Langton.^11^ Their pioneering work on silencing rests on the idea noted earlier, that the successful performance of illocutionary acts requires not only a contribution from the speaker but also a contribution from the audience. The speaker must use a suitable locution (‘There’s a wolf’) with the intention of performing some particular illocutionary act (to *warn* someone of the wolf), while the audience must recognise that that—performing that illocutionary act—is what the speaker is up to. This general dependence of speakers on their audiences for the success of their illocutionary acts makes them vulnerable to a distinctive kind of illocutionary silencing. More specifically, according to Hornsby and Langton, there are certain illocutionary acts which may be rendered ‘unspeakable’ for certain speakers in certain situations, because of factors that interfere with the ability of these speakers’ audiences to give appropriate uptake.

Hornsby and Langton illustrate this idea with the example of sexual refusal. In a social climate where women are seen as likely to ‘play coy’ or where their sincerity is in question, it may be difficult for women to successfully perform the illocutionary act of *refusing* a man’s sexual advances even through the use of seemingly well‐suited locution such as ‘no’. This is because, on account of the prevailing view of women, the audience here may fail to recognise that *refusing* is what she is doing with her words. In this way, sexual refusal becomes ‘unspeakable’ for women in this situation—the woman is not given a say in the sense that her capacity to perform a particular illocutionary act is disabled.

The third sort of silencing is ‘perlocutionary frustration’.^12^ This occurs when an illocutionary act is not disabled but is nonetheless prevented from achieving its desired perlocutionary effect. Of course not all failures to achieve one’s perlocutionary aims should be counted as ‘silencing’ in any meaningful sense. In the classic fable, the boy who cried ‘wolf’ was not silenced when the townspeople remained unconvinced by his warning. Similarly, you do not silence me when you are not amused by my feeble attempts to amuse you. Still, there are cases in which certain speakers are systematically and unjustly blocked from achieving their perlocutionary aims, and it is this kind of case that deserves to be called ‘silencing’.

A leading example of this sort of silencing is the phenomenon that Miranda Fricker calls ‘testimonial injustice’.^13^ Testimonial injustice occurs when a speaker’s act of testimony is given uptake by the audience (the audience recognises what the speaker is up to), but the audience assigns the speaker lower credibility than she deserves because of a prejudicial stereotype about the speaker’s social identity. As a result (at least in typical cases) the audience does not believe what the speaker has told her. In this way, speakers of a certain social type can be subject to a distinctive form of perlocutionary frustration. That is, on account of a prevailing prejudice that impugns their perceived credibility they can be routinely and unfairly prevented from successfully accomplishing the paradigmatic perlocutionary goal of giving testimony, namely, convincing one’s audience to believe what one says. In this sort of case, though the speaker gets a say in the illocutionary sense, her speech is rendered innocuous or inconsequential, and so it is *as though* she has not spoken.

### Group Speech in an Austinian Framework

In the next part of the article, we will be applying this basic Austinian framework for speech acts and silencing to cases of *group speech* and *group silencing*. It might be thought, however, that the sort of acts Austin was talking about are not things that groups have the mental or physical wherewithal to do—and hence that groups can neither speak nor be silenced in any meaningful sense. So before moving on to the application of Austin’s framework to these cases, it may be worth briefly addressing this kind of skepticism about the very possibility of group speech.

We looked earlier at what demands the successful performance of locutionary, illocutionary and perlocutionary acts places on speakers and their audiences. A locutionary act, we saw, is a matter of coming out with a meaningful utterance. At first blush, the idea of a *group* locutionary act might seem puzzling: how could a group, which has no voice or vocal equipment of its own, be capable of saying anything at all—be capable of so much as coming out with words?

On reflection, however, it should be clear that there are a variety of ways that groups can speak with one voice. One way is for the people in the group to coordinate their efforts—to act jointly—in the production of a unified message or utterance. This is what happens, for example, when researchers co‐author research articles; when protestors chant their demands in unison; or when the guests at a birthday party sing ‘happy birthday’ together to their mutual friend. In such cases, each of the group members employs her own linguistic know‐how and apparatus—her understanding of the relevant semantic conventions, and her ability to produce the right noises or signs—to participate in the collective ‘uttering’ of meaningful words. In so doing they jointly produce an utterance that is ascribable to them collectively.

Aside from such collectively‐performed utterances, another central mechanism that groups use for coming out with words—i.e., for group locution—is recruiting an individual person to speak in their collective name. The words of this ‘spokesperson’ are then to be *counted as* the words of the group. Importantly, this form of group locution depends on the spokesperson being properly authorised to speak for the group.^14^

What about group illocutionary acts? Well, an illocutionary act is more than simply coming out with words; it is a distinctive kind of act done with words—such as an act of announcing, promising, warning, and so on. In everyday discourse, such acts are routinely ascribed to groups of various kinds. We say, for instance, that the government *promised* to lower taxes, that the weather bureau *warned* the public of the hurricane, that the commission of inquiry *announced* its findings, and so on. According to our earlier discussion, the performance of such acts typically requires a speaker to perform a suitable locution with a certain kind of communicative intention (which intention the audience recognises, if all goes well). So to make sense of group illocution, it seems we need to make sense of group or collective intentions.

In this connection it is worth noting that several philosophers have developed accounts of group illocution by drawing on the theoretical resources developed in the literature on collective intentionality. For instance, Justin Hughes claims that one of the conditions of group speech acts is that the ‘group has an illocutionary intention’, where this intention is understood in majoritarian or *aggregative* terms: all or most of the group’s members must have the illocutionary intention (and must know of one another that they do).^15^ In contrast to Hughes’s aggregative proposal, Anthonie Meijers has offered a *non‐reductivist* account of group speech according to which group illocutionary intentions (and other collective attitudes) are understood in terms of collective commitments that the group members qua individuals may not share.^16^ More recently, Hans Bernhard Schmid has given an account of group illocutionary intention with reference to the idea of ‘plural pre‐reflective self‐knowledge of what it is we want to say’. On this account it is in virtue of the group members’ having a special kind of self‐knowledge (*plural self* knowledge) that their illocutionary intention is genuinely collective, and hence that they are able to perform group illocutionary acts.^17^

What, finally, about group perlocutionary acts? As we saw earlier, perlocutionary acts are defined in terms of certain distinctive effects that the speaker aims to produce on her audience, such as amusing, persuading, or convincing. These kinds of acts are also routinely ascribed to groups: companies try to persuade customers to buy their latest products; the writers for a sitcom aim to amuse the audience; the legal defence team tries to convince the jury of their client’s innocence, etc. Like physical actions, perlocutionary acts are successful just when an agent successfully affects how things are in the world—in this case, how things are with the audience. So it seems as though to make sense of group perlocutionary acts, we will need some notion of joint action or group agency.

Although group perlocutionary acts have not received much dedicated attention from philosophers, it seems that they too could be accounted for by employing some of the theoretical resources developed in the literature on collective intentionality. For instance, one could adopt Michael Bratman’s individualistic account of shared agency, according to which joint action is the co‐ordinated action of two or more individuals, each of whom has an intention of the form, ‘I intend that we *J*’.^18^ Applying this to the case of group perlocution, *J* would pick out some perlocutionary action (e.g, ‘convince the jury our client is innocent’) and the co‐ordinated action plan would involve different group members playing different but complementary roles (one may give closing statements, one may cross‐examine witnesses, one may keep the files in order, and so on). Alternatively, one could adopt a highly collectivist approach to group action such as Margaret Gilbert’s, according to which joint action is action performed by a ‘plural subject’ and rooted in an irreducible ‘joint commitment’ to which the individuals are parties.^19^ On such an account, a group perlocutionary action would be one that is performed on the basis of a joint commitment to some perlocutionary goal (e.g., ‘amusing the audience’), where this means that each individual owes it to all the others to play her part in their joint fulfillment of that commitment.

Austin’s central insight was that speech is action, and so a fully worked out Austinian account of group speech would need to incorporate an account of the relevant sort of group action. For the purposes of this article, however, we do not need to commit to any particular account. Our main point is simply that there are a variety of established theoretical resources available for developing such an account, and so the idea of group speech is not one that should be dismissed out of hand.

## Group Silencing, Consultation, and Consent

### Collective Rights to Consultation and Consent

We now turn to cases of group silencing that arise in the context of legally‐required consultation with Indigenous or tribal peoples affected by extractive activities such as mining, logging and oil drilling. To properly understand these cases, some background on the rights to consultation and consent might be useful.

Over the past few decades, a right to consultation has been widely recognised in both international law and in many domestic jurisdictions.^20^ Broadly speaking, communities affected by an activity have been recognised as having a right to consultation prior to decisions being taken that affect their land, livelihoods and ways of life. In addition, there has been increasing recognition that Indigenous communities not only have a right to be included in decision‐making processes through consultation, but that in certain circumstances they also have the right to free, prior and informed *consent* (FPIC).^21^

While the exact parameters of these rights differs from one jurisdiction to the next, the United Nations Declaration on the Rights of Indigenous Peoples suggests a number of essential features. First, the rights to consultation^22^ and consent are both recognized as collective rights.^23^ So insofar as consultation and consent involve speech, these rights call for *group speech*: it is these Indigenous communities themselves that are called upon to voice concerns, make assertions, and give or withhold consent with respect to the proposed activities. Second, to realise these rights, Indigenous peoples must be allowed to speak in line with their own traditional decision‐making processes and means of communication.^24^ This means it is up to the community to decide such things as who will speak in their name, which language they will speak, and how far the authority of the spokesperson will extend. Third, the right to consultation places a demand on the state to be at least somewhat receptive to the speech of the community, specifically by taking the information gathered through consultation into consideration in their decision‐making.^25^ The right to consent goes beyond this, placing greater authority in the hands of communities to determine whether a project goes ahead.^26^

The growing recognition of consultation and consent rights for Indigenous communities is certainly a very important legal development, since it promises to address the historical silencing of these communities—the way in which they have been excluded from deliberations and decision‐making processes that affect them.^27^ Yet even when these rights are recognised they may still not be appropriately interpreted or implemented, with the result that Indigenous communities may nonetheless be left vulnerable to practices of silencing. In the remainder of the article, we illustrate this by providing three examples of group silencing—one locutionary, one illocutionary, and one perlocutionary—that take place within the context of consultation and consent practices.

### Locutionary Group Silencing: the Madadeni Dispute

#### Legal context

Our first case study involves the community of Madadeni, in the province of Mpumalanga, South Africa, who have been fighting the licensing of an opencast coal mine on their traditional land.^28^ Under South African law, a mining company cannot be granted a license to prospect, mine or engage in various water‐use activities until it has demonstrated that it has consulted all interested and affected parties, including affected communities who occupy or use the land or have traditional land rights.^29^ Consultation in South Africa, unlike in Latin American countries (discussed in our next examples), is usually carried out by the company seeking a license. In addition to consulting with affected people, the right to consultation includes a right to information about the proposal and the right to have a say and participate in the decision‐making process.^30^

#### Case details

The Madadeni community only became aware of the mining operations after a mining license had already awarded, and excavations on community farmland had begun. From the perspective of community members, it appeared that no consultation process had been conducted, that no information had been provided to them, and that the community had been given no opportunity to raise objections. However, when the community complained that the license had been issued without mandatory community consultation, they were advised by the company that it *had* in fact consulted with them, as it had reached an agreement with their Chief. The Chief, who does not live in Madadeni village, is the traditional leader and representative of the Madadeni community and a number of surrounding communities.

The agreement between the Chief and the mining company was reached after the mining company had arranged certain private payments to the Chief, including purchasing a car for the Chief’s daughter. Nevertheless, the company maintained that because the Chief is the traditional leader of the community, she automatically has the authority to enter into consultation on behalf of the community and to represent the community in any negotiations pertaining to their land, including permitting the company to use community land for mining activities.

In response, the Madadeni community pointed out that, under African customary law, the Chief only has authority to speak for the community if she first engages with the community with respect to the issue at hand.^31^ In other words, the Chief can only act in her capacity as a representative of the community when she follows the collective decision‐making processes prescribed by customary law. In this case, since the Chief did not engage with the wider community and entered into negotiations that benefitted her personally, she failed to speak as a representative of the community.

#### Analysis

This case exemplifies a pernicious consultation practice in which the consulting party attempts to exploit the collective nature of the right to consultation in order to effectively *de‐platform* communities. In South Africa, as in other mining countries, mining and extractive companies have made a habit of selectively engaging with individuals, especially community leaders, who are incentivized to ‘represent’ their communities without the necessary mandate or proper authority.^32^ But garnering the support of improperly authorised representatives is a sly way of circumventing *genuine* community consultation.

In our view, this can be seen as a practice of *locutionary group silencing*, since the group is denied its say in the most basic sense. Recall that the locutionary act is the simple act of coming out with a meaningful utterance, and this is what the community is prevented from doing. In this case, the Chief is indeed a spokesperson for the group, but according to customary law she does not have the authority to represent them until she engages with the community. Since she lacks the proper authority in this instance, her speech is not, in this instance, the speech of the group. But, crucially, because her speech is taken as the group’s, the opportunity for group speech that consultation is meant to secure elapses. Although words are spoken, these are not the words of the group. Their opportunity to have their say has effectively been handed to someone else.

In our view, this deserves to be called ‘silencing’ because it is not just a failure to consult or a procedural misstep on the part of the mining company or the Chief. Instead, it is an active yet subtle way of *de‐platforming* the rightful speaker. The company has taken advantage of the spokesperson mechanism to create the false impression that the community has been consulted, thereby effectively denying them the opportunity to speak. In this way, the community is silenced.

### Illocutionary Group Silencing: *Sarayaku vs Ecuador*

#### Legal context

Our second case study comes from the Inter‐American Human Rights System, which encompasses the Inter‐American Court of Human Rights and the Inter‐American Commission on Human Rights. Over the past few decades, these two institutions have produced a body of important jurisprudence that seeks to advance the rights and interests of Indigenous peoples in the Americas.^33^ The Court and Commission have done this, in part, by recognizing the right of Indigenous peoples to be consulted by their governments about any matter, legislative, administrative or otherwise, that will affect Indigenous communities or their traditional territories. A particularly clear statement of the right to consultation comes from the Court’s judgment in case of *Saramaka People v Suriname*:

the State has a duty to actively consult with [the] community according to their customs and traditions […] This duty requires the State to both accept and disseminate information, and entails constant communication between the parties. These consultations must be in good faith, through culturally appropriate procedures.^34^

What this means is that consultation processes must respect the internal decision‐making processes of the community and its organisations, and be conducted in a culturally appropriate time, place and manner, all of which must be settled in collaboration with the community.^35^

#### Case details

One of the key cases in which the Court focused on Indigenous rights to consultation was the matter of the *Kichwa People of Sarayaku vs the State of Ecuador*.^36^ The dispute was about the State of Ecuador awarding a permit to a private oil company to begin oil exploration activities on traditional Kichwa territory. The Kichwa people staunchly opposed the oil exploration activities and claimed that a number of their rights had been violated in the lead up to the awarding of the permit, including the right to be properly consulted. In its judgment, the Court found in favour of the Kichwa people, in part because the methods of consultation that had been used—methods that included bribes, intimidation, fraud, and ad hoc surveys—did not properly respect the traditional decision‐making structure of the community or its established ways of speaking for itself.

In the course of the proceedings, the Court conducted a site visit. They travelled deep into the Amazon to *themselves* consult with the Kichwa community, ostensibly thereby modeling good consultation practices and giving the community the platform to have their say that had previously been denied them. At a public meeting during the site visit, the *yachak* of the Kichwa people,^37^ Sabino Gualinga, stated that, ‘Sarayaku is a living land, a living jungle; there are trees, medicinal plants and other types of beings there.’ In previous testimony he also made a point of describing different ‘pachas’ of the world, including one at a subterranean level: ‘Beneath the ground, *ucupacha*, there are people living as they do here. There are beautiful towns down there, and there are trees, lakes and mountains.’^38^ The president of the Kichwa, Jose Gualinga, emphasized the importance of the forest to the community, claiming that ‘[the forest] gives us the power, potential and energy that is vital to our survival and life. And everything is interconnected with the lagoons, the mountains, the trees, the beings and also us as an exterior living being.’^39^

These claims were made by authorised spokespersons for the Kichwa people, who were being called upon by the Court to speak in the name of their community. What is more, their claims were made in the context of explaining the Kichwa people’s opposition to oil exploration activities that would involve drilling under the ground and destroying parts of the forest. Given this context, and the fact that these claims have the surface structure of typical assertions, it seems natural to interpret them as the Kichwa people’s assertions about the state and nature of the environment. Yet, strikingly, the Court did not appear to hear these claims in this way. This is suggested by the way that it summed up the section of its judgment in which these claims are featured. Without appearing to evaluate the truth of those claims—without accepting or denying that there are mountains and lakes under the ground, that everything in the forest is interconnected, etc.—it simply concludes that:

the Kichwa people have a profound and special relationship with their ancestral territory, which […] encompasses their own worldview and cultural and spiritual identity.^40^

It seems to us that the Kichwa people were attempting to make straightforward assertions about their natural environment, but were heard as making claims of an altogether different sort—claims about their ‘worldview’ and ‘cultural and spiritual identity’. So it is not that the Court recognized that the community meant to make assertions about their natural environment, but simply dismissed these assertions as false. On the contrary, the Court did not seem to give those claims the epistemic attention that assertions typically demand: it neither accepted not rejected them, nor did it take them to bear evidentially on related issues, such as the environmental impacts of the proposed oil drilling.^41^ Moreover, the way the Court presented its conclusion that ‘the Kichwa people have a profound and special relationship with their ancestral territory, which […] encompasses their own worldview and cultural and spiritual identity’ is very much in the spirit of *agreement* (or acceptance), as a kind of re‐statement of what, according to the Court, the Kichwa themselves were saying.

It is also instructive to note that this reception of the community’s speech stands in marked contrast to the way the Court treated the speech of other parties called upon to give evidence, such as expert witnesses. For instance, when an environmental engineering expert, William Powers, described in his expert report the likely impacts of a large oil project in the forest, including the clearing of vegetation, impacts on water courses, soil erosion, etc, these claims were not taken by the court as expressive of William Powers’ ‘worldview’, but simply as straightforward assertions about the likely impacts of the proposed activities on the environment in question. ^42^ However, when the Kichwa people attempted to make similarly straightforward claims about their environment, these claims were seen by the Court as expressive of the Kichwa’s worldview or cultural and spiritual identity, rather than as assertions about the environment.^43^

#### Analysis

The Court’s interpretation of Indigenous testimony in this case exemplifies another problematic practice frequently seen in community consultation processes. In this case the issue is not that of de‐platforming, since every effort is made to ensure that the community is given an opportunity to speak, in line with its traditional decision‐making procedures and modes of representation. Instead, the issue concerns the *reception* of the community’s speech. That is, the words of the community appear not to be taken as the community means them to be taken, with the result that the community is prevented from ‘having its say’ in the communicative sense. More specifically, the Kichwa community tries to perform the illocutionary act of *asserting* about the state of their environment, using the right words (i.e., locutions fit for the purpose) in the right mouths (i.e., the mouths of properly authorized representatives). Yet, because of the way these words are taken up by the Court—not as assertions about the environment but as expressions of the community’s ‘worldview’—the community finds it is unable to successfully perform those assertions. Though they are allowed to speak in the locutionary sense, their *illocutionary* potential is hampered.

We think this practice can be usefully understood as a form of the ‘illocutionary disablement’ discussed by Hornsby and Langton. Recall that, for Austin, one of the necessary conditions for the successful performance of any illocutionary act is that the audience recognises the act as it is intended by the speaker. What Hornsby and Langton demonstrated was that widely‐held misconceptions can seriously impede the illocutionary capacity of women in sexual contexts, by systematically interfering with the ability of their audiences to recognise their illocutionary acts of refusal. As a result, women’s attempts to refuse sex are silenced—the speech act of refusal becomes ‘unspeakable’ for women in this context.

In a similar way, we think that widely‐held prejudicial views about the epistemic credentials and authority of Indigenous communities may systematically impede the illocutionary capacities of these communities. Such prejudicial views are espoused within judicial institutions. For example, as Rebecca Tsosie has noted, ‘Courts are unlikely to recognise tribal members as having the same credibility as an “expert witness” [… and] the categories of knowledge that cultural practitioners hold are often invisible with the US legal system.’^44^ Our suggestion is that, because Indigenous communities are not seen as having the epistemic authority to make claims about such things as the state of the natural environment, their attempts to do so are routinely not recognised as such—they fail to secure ‘uptake’.^45^ In this way, assertions about the natural environment become ‘unspeakable’ for these communities in this context. This is illocutionary group silencing.

### Perlocutionary Group Silencing: Colombia’s Footnotes to the ADRIP

#### Legal context

The right to consultation, discussed in the previous two case studies, is a procedural participatory right. It requires decision‐makers to follow certain procedures (to consult with communities) and to take certain information into account in decision‐making (submissions and statements made by those communities in the course of the consultation process). It is not a right that determines the outcomes of the decision‐making process; it does not guarantee communities continued occupation of their territories nor does it grant them the right to refuse mining companies access to their land. For this reason, many see the right to consultation as too weak to meaningfully protect Indigenous communities, and as a result Indigenous rights activists have argued that the state should not simply consult but also obtain the free, prior and informed *consent* (FPIC) of the community before making decisions that will impact its land and livelihood.^46^ As a consequence of these efforts, the right to FPIC has now been recognized in international legal texts^47^ and by the Inter‐American Court of Human Rights.^48^

Our final case study relates to the right to FPIC. Although often undefined in law, the right to FPIC is understood as something that goes beyond consultation—it does not merely give Indigenous communities a say, but is meant to dramatically *empower* their speech, giving them the special standing to permit or refuse development on their land.^49^ However, as our case study illustrates, some states have sought to neutralise the impact of the right to FPIC, by rendering the *withholding* of consent inconsequential.

#### Case details

While there is growing recognition of the right of Indigenous peoples to FPIC in both international and domestic law, a number of states have interpreted this right in such a way as to effectively neutralise the consent requirement.^50^ In particular, they have argued that while Indigenous communities do have the right to consent or withhold consent to developments on their territories, they do not have the power of ‘veto’ over whether such developments go ahead.^51^ These states are seeking to ensure that the withholding of community consent does not prevent development activities on Indigenous territories.

A clear example of this can be seen in the stance taken by Colombia to the recently adopted American Declaration on the Rights of Indigenous Peoples (ADRIP).^52^ The declaration states that

[member states must] obtain the free, prior and informed consent [of affected Indigenous peoples] before adopting or implementing legislative or administrative measures that may affect them^53^ […and] before approving projects that could affect their lands or territories and other resources.^54^

Colombia, a signatory to the declaration, added the following ‘footnotes’ in regard to the consent requirement:

[the consent requirement] does not translate into the ethnic communities having the power of veto over measures affecting them directly whereby such measures cannot proceed without their consent; instead it means that following a disagreement “formulas for consensus‐building or agreement with the community” must be presented.^55^

In other words, Colombia is prepared to allow Indigenous communities to withhold their consent, but their doing so will not mean that the proposed activities do not go ahead. Instead, when the community withholds its consent, this will simply occasion further negotiation and ‘consensus‐building’ activities.

#### Analysis

Colombia’s approach to the right to consent is problematic because it renders the withholding of consent innocuous. It stands in contrast with the way rights to consent are commonly understood. The idea of FPIC originates in the field of medical ethics^56^ where a competent patient’s decision to give or withhold consent is meant to settle the matter of whether the medical procedure will proceed. While it might, in some circumstances, be appropriate for a medical professional to try to convince a patient to proceed with an operation, a patient’s giving or withholding of consent is meant to be determinative of whether the procedure will take place. In this context the withholding of consent is meant to convince the other party to stop. This is exactly how the UN Human Rights Commission has defined FPIC in the context of Indigenous rights:

An affirmative “no” or “withholding of consent” expresses the Indigenous peoples’ opposition to the project and is expected to convince the other party not to take the risk of proceeding with the proposal.^57^

While there may be circumstances in which a state can engage in further discussions in regard to whether a proposal might be acceptable to a community who has withheld its consent,^58^ Colombia’s footnotes suggest that a community’s withholding of consent will not (as a matter of law) convince the state to stop a project or proposal.

We think this amounts to a form of what could be called ‘perlocutionary frustration’. Perlocutionary acts are those things speakers accomplish *by* their speech. So if I tell you something, where telling is an illocutionary act, then I might *thereby* persuade you to believe what I say—where this, persuading someone of something, is a perlocutionary act. Similarly, consenting and withholding consent (or refusing) are illocutionary acts, but there are perlocutionary aims that speakers tend to use them for. Specifically, when one consents one typically means to allow another party to go ahead with some proposed activity, and when one withholds consent one intends to convince the other party to stop some proposed activity from going ahead. But what we see in the case of Colombia’s footnote to the Declaration is that this perlocutionary aim of withholding consent—to convince the other party to stop some activity from going ahead—is systematically forestalled. In effect, Colombia is saying that when it comes to the community’s consent or refusal, they will not take ‘no’ for an answer. More precisely, they will never allow the illocutionary act of refusal to accomplish its standard perlocutionary aim. So while the Indigenous community is granted the opportunity to speak, and while it will be recognised by the state as performing the illocutionary acts of granting or withholding consent, its speech is nonetheless rendered utterly inconsequential. This is perlocutionary group silencing.

## Conclusion

Historically, Indigenous peoples have been denied a say in decisions about their lands and livelihoods. The growing recognition of Indigenous rights to consultation and consent in international and domestic law has sought to recognise the sovereignty of Indigenous peoples and to ensure their participation in those decisions. However, we have argued that the interpretation and implementation of these rights has meant that communities are still routinely silenced within these decision‐making processes.

To illustrate this, we have examined three cases in which, we argue, Indigenous communities have been silenced despite the appearance of a fair participatory process. Using the theoretical framework of feminist speech act theory, and in particular the Austinian approach to silencing developed by Hornsby and Langton, we have illuminated three ways in which Indigenous communities can be silenced *as group speakers* in participation processes. *Locutionary group silencing* occurs when the platform for group speech is occupied by someone who lacks the proper authority to speak for the group, and so the opportunity for group speech elapses. *Illocutionary group silencing* occurs when a group’s attempts to perform certain speech acts are not given appropriate uptake, and so fail. And *perlocutionary group silencing* occurs when the perlocutionary aims of a group’s speech are systematically blocked.

This investigation of practices of group silencing in community consultation has both philosophical and legal value. From a philosophical perspective, while questions about the possibility and nature of group speech have been gaining increased attention in recent years, questions about the politics of group speech have been roundly neglected. In light of the substantial role played by collective bodies—governments, institutions, communities, etc—in public discourse, this is a serious lacuna in the literature. Highlighting practices in which the speech of certain groups is disempowered helps to bring these neglected questions into view, while the framework of feminist speech act theory helps to identify certain distinctive ways in which group speech can be disempowered.

From a legal perspective, the value of applying this framework for thinking about speech acts and silencing is that it reveals how Indigenous communities can be denied a proper say even when they have been ‘included’ in deliberations and decision‐making. In this way, it tells us something about the requirements of truly meaningful consultation and consent. While rights to consultation and consent are meant to ensure Indigenous communities get a say in decisions that affect them, if these rights are not appropriately implemented and interpreted, they can fail to give Indigenous communities a proper say, and succeed only in silencing and marginalising them further.^59^

## Notes

1 In many parts of the world, colonial powers sought to justify their ‘ownership’ of colonized land via the ‘doctrine of discovery’—the notion that prior to colonialism this land was not owned or occupied and hence could be acquired through occupation. Indigenous peoples were not recognised as occupiers or owners of the land, nor were they seen to have any entitlement to determine what happened to the land or the resources found on that land. Indigenous peoples were denied their autonomy, sovereignty and, often, their very humanity was not recognised. See https://www.un.org/press/en/2012/hr5088.doc.htm

2 See, ILO, *Indigenous and Tribal Peoples Convention, C169*; *UNGA Declaration on the Rights of Indigenous Peoples (2007) (UNDRIP)*; Organisation of American States, *American Declaration on the Rights of Indigenous Peoples* (2016) (ADRIP).

3 Countries as diverse as Honduras, South Africa, and Norway have all adopted laws on Indigenous participatory rights.

4 JL Austin, *How to Do Things With Words* (Oxford: Oxford University Press, 1962).

5 See esp. Jennifer Hornsby, ‘Illocution and its significance’ in S. L. Tsohatzidis (ed.) *Foundations of Speech Act Theory: Philosophical and Linguistic Perspectives* (Abingdon: Routledge, 1995): 187–207; Jennifer Hornsby, ‘Disempowered speech’, *Philosophical topics* 23, 2 (1995): 127–147; Rae Langton, ‘Speech acts and unspeakable acts’, *Philosophy & Public Affairs*, 1993: 293–330.

6 Austin op. cit., p. 116‐117.

7 Langton op. cit.

8 In addition to Hornsby and Langton’s broadly Austinian approach to silencing, other accounts of silencing have been developed within other speech‐act‐theoretic frameworks. This includes the Gricean approach of Ishani Maitra (‘Silencing speech’ *Canadian Journal of Philosophy* 39, 2 (2009): 309‐338.), and the normative functionalist approach of Rebecca Kukla (‘Performative Force, Convention, and Discursive Injustice’ *Hypatia* 29, 2 (2014):440‐457). Because of the distinction it draws between three distinctive forms of silencing, we find the Austinian approach the most illuminating for the purposes of this paper. For a discussion of how Maitra’s and Kukla’s approaches to silencing might be applied to the silencing of Indigenous speech, see Leo Townsend, ‘Group assertion and group silencing’, *Language & Communication* 70, 1 (2020): 28‐37.

9 Langton op. cit., p.315.

10 Langton op. cit., p.315.

11 See Jennifer Hornsby and Rae Langton, ‘Free speech and illocution’, *Legal Theory* 4, 1 (1998): 21–37; Hornsby, ‘Illocution and Its Significance’ op. cit.; Hornsby, ‘Disempowered Speech’ op. cit.; Langton op. cit.

12 Langton op. cit., p.315.

13 Miranda Fricker, *Epistemic Injustice: Power and the Ethics of Knowing* (Oxford: Oxford University Press, 2007), chap. 1.

14 For two recent discussions of the role of spokespersons in group speech, see Kirk Ludwig, ‘Proxy agency in collective action’, *Noûs* 48, 1 (2014): 75–105; Jennifer Lackey, ‘Group assertion’, *Erkenntnis* 83, 1 (2018): 21–42.

15 Justin Hughes, ‘Group speech acts’, *Linguistics and Philosophy* 7, 4 (1984): 379–395.

16 Anthonie Meijers, ‘Collective speech acts’ *Intentional acts and institutional facts* (Dordrecht: Springer, 2007): 93–110.

17 Hans Bernhard Schmid, ‘Groups speaking for themselves: Articulating first‐person plural authority’, *Language & Communication* 70, 1 (2020): 38‐45.

18 Michael Bratman, ‘I Intend That We J’ *Faces of Intention: Selected Essays on Intention and Agency* (Cambridge: Cambridge University Press, 1999): 142–161.

19 Margaret Gilbert, *Joint Commitment: How We Make the Social World* (Oxford: Oxford University Press, 2013).

20 See footnotes 2, 3.

21 The UNDRIP refers to consent in articles 10, 11, 19, 28, 29, and 32. Although not binding, the UNDRIP has been signed by about 150 countries and its “norms have become the basis for an international consensus about Indigenous rights, and […] have begun to diffuse to national and local level.” Jason Tockman, ‘Eliding consent in extractivist states: Bolivia, Canada, and the UN Declaration on the Rights of Indigenous Peoples’, *The International Journal of Human Rights* 22, 3 (2017), p. 325. Consent standards have also been incorporated by the World Bank in its 2016 Environmental and Social Standards.

22 Consultation is recognized as both an individual and a collective right.

23 See article 1

24 See article 19, article 30(2) and article 32(2).

25 This is implied in the notion of ‘good faith’ in articles 19, 32(2) and (3).

26 Tara Ward, ‘The Right to Free, Prior, and Informed Consent: Indigenous Peoples’ Participation Rights within International Law’, *Northwestern University Journal of International Human Rights* 10 (2011): 54–84, at p. 54.

27 See the discussion of the importance of consultation to indigenous participation in public affairs and governance in the Inter‐American Commission Report “Indigenous Peoples, Afro‐Descendent Communities, and Natural Resources: Human Rights Protection in the Context of Extraction, Exploitation, and development activities" 2016).

28 This is an ongoing case in which one of us, Dina Lupin Townsend, was previously involved as an attorney representing the community. Information on this case is taken from the case notes of the attorneys for the community, the Centre for Environmental Rights (CER) and is used with the kind permission of the Madadeni Mining Committee and the CER.

29 Rights to consultation fall under different pieces of legislation, including the Mineral and Petroleum Resources Development Act, 2002, and the National Water Act, 1998.

30 See *Bengwenyama Minerals (Pty) Ltd and Others v Genorah* *Resources (Pty) Ltd and Others *2011 (4) SA 113 (CC). Companies are required to carry out consultation through an environmental impact assessor.

31 African Customary Law is recognised source of law under the Constitution. See Chuma Himonga and Craig Bosch, ‘The Application of African customary law under the constitution of south Africa: Problems solved or just beginning’, *South African Law Journal* 117 (2000): 306.

32 Another example of this is found in the case we discuss in the next section, *Kichwa Indigenous People of Sarayaku v Ecuador*, 2012. In that case, the company attempted to bribe individuals within the community to agree to drilling on Indigenous territory. In another recent mining controversy in South Africa, affecting the Xolobeni community, the mining company allegedly bribed chiefs with offers of 4x4 vehicles. See ‘Corruption Impeding on Human Rights in South Africa’s Mining Sector’, *IACC Series*, <https://iaccseries.org/blog/corruption‐impeding‐on‐human‐rights‐in‐south‐africas‐mining‐sector/> [accessed 4 October 2019].

33 For an overview of key cases see Inter‐American Commission on Human Rights, op. cit note 27.

34
*Saramaka People v Suriname*, 2007.

35 Inter‐American Commission on Human Rights, op. cit note, paras 207‐212. See also Secretariat of the Convention on Biological Diversity, ‘Akwé:Kon Guidelines’ (2004).

36
*Sarayaku v Equador* op. cit.

37 A yachak (literally ‘one who knows’) is a spiritual leader or shaman.

38
*Sarayaku v Equador* op. cit., para.150.

39
*Sarayaku v Ecuador* op. cit., para.152.

40
*Sarayaku v Ecuador* op. cit., para.155.

41 For example, while the Court considers this testimony to be relevant to *social* impact assessments, it appears to see no relevance for the environmental impact assessment. See Dina Lupin Townsend, ‘Silencing, consultation and indigenous descriptions of the world’ *Journal of Human Rights and the Environment* 10, 2 (2019): 193‐214.

42
*Sarayaku v Ecuador* op. cit., para.174.

43 Of course, it is possible that the community here is not attempting to make straightforward assertions. That is, it could be engaging in ‘mythical discourse’ rather than fact‐stating discourse, and it may be that there is no clear distinction between these within their linguistic practice (cf. Rebecca Tsosie, ‘Indigenous Peoples, Anthropology, and the Legacy of Epistemic Injustice’ *The Routledge Handbook of Epistemic Injustice* (Routledge, 2017): 356–369.)

44 Rebecca Tsosie ‘Indigenous peoples and epistemic injustice: Science, ethics, and human rights. *Washington Law Review* 87 (2012): 1133‐1201.

45 It is worth noting that in some jurisdictions there has been important progress in recognizing Indigenous forms of knowledge and evidence—see, e.g., the case of *Delgamuukw v British Columbia* (1997, Canadian Supreme Court), in which the oral history of Indigenous communities was recognised as evidence. (We thank an anonymous reviewer for pointing out the relevance of this case.)

46 See e.g. Riccarda Flemmer and Almut Schilling‐Vacaflor, ‘Unfulfilled promises of the consultation approach: the limits to effective Indigenous participation in Bolivia’s and Peru’s extractive industries’, *Third World Quarterly* 37, 1 (2016): 172–188; Amanda Fulmer, ‘The politics of a strange right: consultation, mining, and Indigenous mobilization in Latin America’ in G. Andreaopoulos and Z. F. Kabasakal Arat (eds.) *The Uses and Misuses of Human Rights* (Springer, 2014): 65–88; César Rodríguez‐Garavito, ‘Ethnicity. gov: Global governance, Indigenous peoples, and the right to prior consultation in social minefields’, *Indiana Journal of Global Legal Studies* 18, 1 (2011): 263–305.

47 See note 21.

48
*Sarramaka v Suriname* op. cit.

49 ILO Convention 169 op. cit art 6; Ward op. cit 56.

50 Note that while this is commonly referred to as the right to free, prior, and informed consent, it is often defined as a duty on the State to *obtain* consent (see e.g., Article 19 of ADRIP). Hence it might be more accurately described as a right of Indigenous peoples to *withhold* consent. (Thanks to an anonymous reviewer for suggesting we clarify this.)

51 The argument that the right to consent does not amount to a right to ‘veto’ is a common one in extractive states, including in Bolivia and Canada. See the discussion in Tockman op. cit.

52
*ADRIP* op. cit.

53 Article XXIII(2).

54 Article XXIX(4).

55 UNDRIP op. cit., footnote 3. See also ADRIP footnote 4.

56 Anne Perrault, ‘Facilitating prior informed consent in the context of genetic resources and traditional knowledge’, *Sustainable Development Law & Policy* 4 (2004): 21, at p. 21.

57 HRC, Study of the Expert Mechanism on the Rights of Indigenous Peoples on FPIC, 2018, para 25.

58 The UNDRIP distinguishes between circumstances in which states must ‘have consent as the objective of consultation’ (articles 19 and 32) and circumstances in which the state is obliged to *obtain* the consent of the community concerned (article 10 and 29). The ADRIP does not make this distinction, requiring only that states obtain consent.

59 We are grateful to audiences in Trieste, Boston and Vienna, as well two anonymous reviewers for this journal, for their generous feedback. Leo Townsend also gratefully acknowledges the support his research receives from the Austrian Science Fund (FWF), grant number I‐3068‐G24.

